# Consistent and reproducible cultures of large-scale 3D mammary epithelial structures using an accessible bioprinting platform

**DOI:** 10.1186/s13058-018-1045-4

**Published:** 2018-10-10

**Authors:** John A. Reid, Peter M. Mollica, Robert D. Bruno, Patrick C. Sachs

**Affiliations:** 1 Biomedical Engineering Institute, College of Engineering, Old Dominion University, 5115 Hampton Blvd, Norfolk, VA 23529 USA; 2 School of Medical Diagnostic & Translational Sciences, College of Health Sciences, Old Dominion University, 5115 Hampton Blvd, Norfolk, VA 23529 USA

**Keywords:** Bioprinting, 3D culture, Organoids, Mammary epithelium, Tissue engineering, Regenerative medicine, Biofabrication

## Abstract

**Background:**

Standard three-dimensional (3D) *in vitro* culture techniques, such as those used for mammary epithelial cells, rely on random distribution of cells within hydrogels. Although these systems offer advantages over traditional 2D models, limitations persist owing to the lack of control over cellular placement within the hydrogel. This results in experimental inconsistencies and random organoid morphology. Robust, high-throughput experimentation requires greater standardization of 3D epithelial culture techniques.

**Methods:**

Here, we detail the use of a 3D bioprinting platform as an investigative tool to control the 3D formation of organoids through the “self-assembly” of human mammary epithelial cells. Experimental bioprinting procedures were optimized to enable the formation of controlled arrays of individual mammary organoids. We define the distance and cell number parameters necessary to print individual organoids that do not interact between print locations as well as those required to generate large contiguous organoids connected through multiple print locations.

**Results:**

We demonstrate that as few as 10 cells can be used to form 3D mammary structures in a single print and that prints up to 500 μm apart can fuse to form single large structures. Using these fusion parameters, we demonstrate that both linear and non-linear (contiguous circles) can be generated with sizes of 3 mm in length/diameter. We confirm that cells from individual prints interact to form structures with a contiguous lumen. Finally, we demonstrate that organoids can be printed into human collagen hydrogels, allowing for all-human 3D culture systems.

**Conclusions:**

Our platform is adaptable to different culturing protocols and is superior to traditional random 3D culture techniques in efficiency, reproducibility, and scalability. Importantly, owing to the low-cost accessibility and computer numerical control–driven platform of our 3D bioprinter, we have the ability to disseminate our experiments with absolute precision to interested laboratories.

**Electronic supplementary material:**

The online version of this article (10.1186/s13058-018-1045-4) contains supplementary material, which is available to authorized users.

## Background

Three-dimensional (3D) culture systems for generating organoid cultures of mammary epithelial cells inside collagen matrices were introduced over four decades ago [1]. In 3D culture, multiple parameters operate together to affect both experimental outcomes and interpretation of experimental results. These parameters include cell type, cell–cell interactions, extracellular matrix (ECM) composition, culture media, and mechanical properties such as matrix stiffness and cell confinement [2–12]. Standard 3D culture procedures involve either mixing dispersed mammary epithelial cells within ECM substrates prior to gelling or culturing cells on top of a pre-formed ECM gel. Once polymerized, the ECM gel can also be left attached to the culture dish or floated. The encapsulated cells will randomly organize into organoids which remodel and reorganize the substrate matrix to generate structures composed of morphologically polarized cells facing an open lumen [13–17]. However, the size and morphology of resulting organoids vary greatly, even within the same ECM gel substrate. While some variability inevitably results from disparities in local environmental conditions, such as collagen fiber anisotropy within specific regions of a gel, a major source of potentially controllable variability results from the random distribution of cells within the gel [18–23]. This variability leads to difficulty in interpreting and reproducing results, especially from laboratory to laboratory. As inter-laboratory reproducibility is a major concern in modern biomedical research, platforms that will allow better control and reproducibility are highly desired [24].

Recent advances in material science have promoted the development of novel synthetic hydrogels with tunable physiochemical properties for cell encapsulation and 3D bioprinting of mammary epithelial cells [25]. However, layer-by-layer methods for 3D cell printing frequently require extrusion nozzles near 300 μm and layer thicknesses of 500 μm, which severely limit the ability to control aspects of the microenvironment at the single-cell scale. We recently described the adaptation of an off-the-shelf 3D printer for the purposes of bioprinting cells within precast 3D substrates [26]. This system uses pulled glass microneedles, which can be designed with tip diameters ranging from 10 to 100 μm, allowing more accurate cell placement down to the single-cell level. We have previously established that multiple needle insertions into a polymerized collagen gel did not disrupt neighboring cell deposits [26]. The glass microneedles used in our system are non-coring and thin enough to allow the gel to seal behind them when removed. Furthermore, because our extrusion apparatus is designed to reliably handle volumes of less than 1 nL, it is unlikely that this volume would significantly alter the local microenvironment associated with a fibrous scaffold, such as collagen. Our overall goal was to design an accessible, open-access bioprinter that would not be cost-prohibitive to research laboratories. Because of the precision afforded by the computer numerical control (CNC) system and the ability to share the G-Code underlying the printing process, the use of bioprinting in basic research laboratories offers promise for new standards designed to increase internal and intra-laboratory experimental reproducibility. Specifically, the use of CNC systems to control the spatial deposition of cells in 3D structures appears well suited to recreate the tissue-specific contextual cues needed to overcome the limitations of manual pipette patterning [26, 27]. Here, we describe the adaptation and validation of our accessible bioprinter to produce high-fidelity, spatially controlled arrays of human mammary organoids inside 3D collagen matrices. We demonstrate the superiority of our printing process over manual matrix embedding techniques in efficiency and consistency in organoid morphology. We further describe parameters necessary to generate large luminal organoids with shapes dictated by print locations (for example, linear or circular). These data lay the groundwork for adaptation to investigate additional cell types and 3D matrices, thereby providing an ideal method to derive empirical standards aimed to improve the *in vitro* culture of biological processes such as development and tumorigenesis.

## Methods

### Cell culture

Immortalized non-tumorigenic human breast epithelial cell lines MCF12A and MCF10A were purchased from the American Type Culture Collection (Manassas, VA, USA). MCF12A and MCF10A cells were initially cultured in 2D on tissue culture plastic in a 75-cm^2^ flask supplemented with a 1:1 mixture of Dulbecco’s modified Eagle’s medium and Ham’s F12 medium (DMEM/F12), 5% Horse Serum, 20 ng/mL human epidermal growth factor (hEGF), 0.01 mg/mL bovine insulin, 500 ng/mL hydrocortisone, and 1% ABAM (all purchased from Thermo Fisher Scientific, Waltham, MA, USA). Cells were cultured at 37.0 °C and 5.0% carbon dioxide (CO_2_). After confluence, the cells were dissociated using TrypleE (Thermo Fisher Scientific) and collected by centrifugation.

### Preparation of ECMs and manual cell-matrix embedding

For manual cell-matrix embedding studies, single-cell suspensions of MCF12A or MCF10A cells were mixed with neutralized rat tail collagen I (Corning, Corning, NY, USA) as specified by the manufacturer, unless noted otherwise, to a final concentration of 1.5 mg/mL. Immediately after mixing, 500 μL of neutralized collagen I gel material, containing about 5000 cells, was dispensed into a 24-well plate and allowed to solidify and adhere to the surfaces of the well for 1 h in a laboratory incubator at 37.0 °C and 5.0% CO_2_. After gelation (solidification), 500 μL of cell media was added to the wells. Subsequent media changes were performed every 3 days. VitroCol, human collagen I solution (Advanced BioMatrix, San Diego, CA, USA), was prepared in accordance with the recommendations of the manufacturer to a final concentration of 1.0 mg/mL. Hydrogels of growth factor–reduced, LDEV-free Matrigel (Geltrex; Thermo Fisher Scientific) were prepared at 37 °C using the stock solution without dilution in accordance with the protocol of the manufacturer. For all printing experiments, a minimum of 500 μL of collagen gel was dispensed into individual wells of a 24-well plate and allowed to solidify for 1 h in a laboratory incubator at 37.0 °C and 5.0% CO_2._ For all experiments, cells were monitored by using a combination of bright-field imaging/fluorescent imaging using a Zeiss axio-observer Z1 fluorescent microscope (Carl Zeiss AG, Oberkochen, Germany) or time-lapse imaging using a Lumascope 620 microscope (Etaluma, Carlsbad, CA, USA).

### Bioprinting system

A previously developed bioprinting system was used to robotically insert a microneedle into specified 3D locations of a polymerized collagen gel [26]. Immediately before printing, 2D cultures of MCF12A cells were dissociated into single cells using TrypleE (Thermo Fisher Scientific), centrifuged at 300*g*, and re-suspended in media to obtain a final “ink” concentration of 60 × 10^4^ cells per milliliter. Shortly thereafter, 50 μL of cell-containing “ink” was loaded into a sterile needle. Printing operations were initiated after the “ink”-containing needle was attached to the print head. The number of cells deposited in a target location was manipulated by varying the volume of cell-containing “bio-ink” extruded from the needle tip, or to equalize volumes, by increasing or decreasing initial cell concentration. Printing operations were optimized to extrude specified numbers of cells with a volume per print of less than 1 nL inside the collagen I gel via a CNC insertion routine which deposited cell-containing media at a specified “target” location inside the polymerized collagen I gel. Users specified intended wells of commercially available tissue culture plates, printing locations, distances among printing locations, and the number of cells per target location. The experiment information was automatically converted into G-Code, loaded into Repetier Host (www.repetier.com), and sent to the three-axis microcontroller of the bioprinter. The bioprinting system was located inside a benchtop biosafety cabinet during all printing operations. The heated print bed was set to 37 °C for all printing operations. Needles used by the bioprinting device were fabricated by using a Sutter P97 programmable pipette puller to have tip diameters of 50 μm. All printing equipment was sterilized by using a steam autoclave prior to printing procedures. After printing routines were complete, plates were covered with 500 μL of media and placed inside a laboratory incubator at 37 °C, 5% CO_2_. After printing, cells were monitored by using a combination of bright-field imaging/fluorescent imaging using a Zeiss axio-observer Z1 fluorescent microscope or time-lapse imaging using a Lumascope 620 microscope (Etaluma). Cell-specific media exchange was performed every 3 days. All experimental conditions were performed in triplicate.

### Characterization of organoid growth and morphology

Immediately after printing, the initial quantity of printed cells was verified by using manual counting and image analysis using ImageJ and Matlab. After printing, cells were monitored up to 21 days by using a Zeiss axio-observer Z1 fluorescent microscope. The size of organoids was determined by analyzing bright-field images taken daily for each experimental condition using ImageJ. Within this investigation, organoids were operationally defined as a cluster of cells with no clear cell–cell boundaries or the inability to discern individual cells from neighboring cells. All experiments were performed a minimum of three separate times. All quantitations presented in the results represent total observations across at least three independent experiments.

### Immunofluorescence staining

Gels were fixed in 10% neutral buffered formalin, paraffin-embedded, and sectioned. Sections were prepared for staining by deparaffinizing in a xylene substitute, rehydration, and heat-mediated antigen retrieval using pH 9 tris-EDTA with 0.05% tween 20. Sections were blocked in 10% goat serum and incubated with primary antibodies in a humidified chamber at 4 °C overnight. Secondary antibodies were added for 1 h at room temperature. Sections were counterstained with 4′,6-diamidino-2-phenylindole (DAPI). Antibodies were used at the following concentrations: anti-green fluorescent protein (anti-GFP) rabbit IgG Alexa Fluor 488 conjugated (1:75; Invitrogen A21311, Invitrogen, Carlsbad, CA, USA), rabbit polyclonal antibody to GJB1 [Cx32] (1:25; HPA010663, Sigma-Aldrich, St. Louis, MO, USA), biotinylated rabbit polyclonal antibody to red fluorescent protein (RFP) (1:500; Abcam, Cambridge, UK, ab34771), rabbit anti-laminin 1 + 2 (1:100; Abcam, ab7463), and mouse anti-laminin 5 antibody (1:100; Abcam, ab78286), Alexa Fluor 488 and 568 conjugated goat secondary antibodies (1:1000; Thermo Fisher Scientific) or avidin Alexa Fluor 488 (Thermo Fisher Scientific A21370). All sections were counterstained with DAPI and imaged by using a Zeiss axio-observer Z1 fluorescent microscope.

## Results

### Generation of consistent individual mammary epithelial organoids

**Fig. 1:**
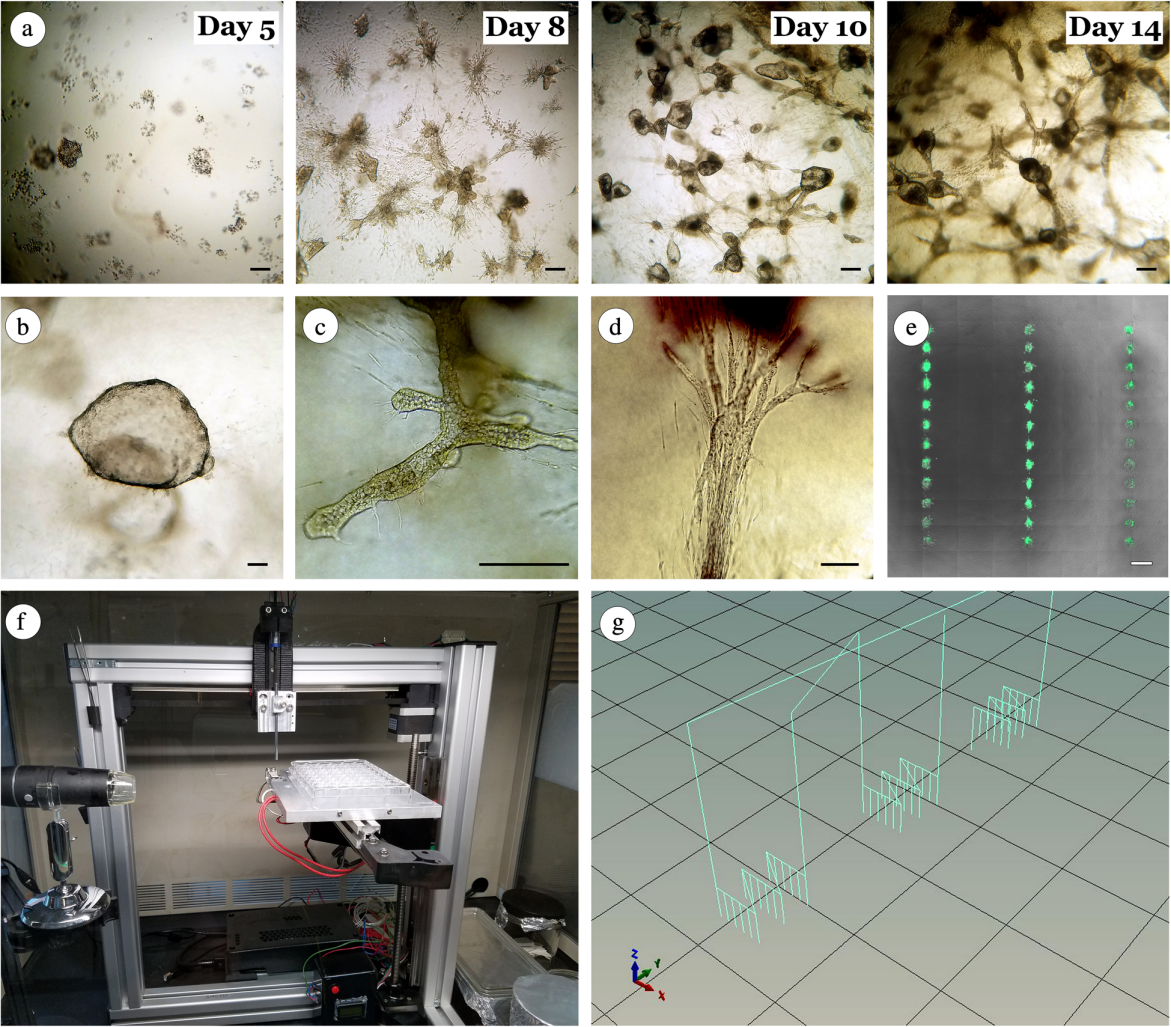
Manual matrix embedding versus bioprinting of MCF12A cells. (**a**) Example of random organoid dispersion and morphology of MCF12A cells following manual matrix embedding in collagen gels at 5, 8, 10, and 14 days. (**b–d**) Examples of resulting organoid morphologies from manual matrix embedded MCF12a cells: (**b**) “sphere-like”, (**c**) “duct-like”, (**d**) “star-like”. (**e**) Example of controlled and organized growth of MCF12A organoids following three-dimensional (3D) bioprinting. (**f**) Image of our accessible bioprinting platform. (**g**) Visual representation of machine path during insertion routine*.* Scale bars: **a** = 200 μM, **b–d** = 100 μM, **e** = 500 μM

As the main aim of this work was to validate experimental bioprinting methods for investigating mammary epithelial biology in 3D culture, we first established baseline behaviors of MCF12A cells using manual cell-matrix embedding techniques. Similar to previous findings [21, 28], we found an extremely high level of inter- and intra-experimental variability, which appears to be mostly independent of culturing conditions. Day 1 after the manual embedding of cells within collagen gels, the cells were similar in morphology, and cells either remained dispersed individually or formed small clusters (data not shown). This activity seemed to correspond with the random nature in which the cells were embedded in the gel—that is, whether cells ended up in close proximity to other cells or not. Noticeable structures emerge by day 5, and by day 8 prominent structural variations appear that by day 14 establish into distinguishable organoids (Fig. 1a). Three common organoid morphologies were observed—sphere-like (Fig. 1b), duct-like (Fig. 1c), and star-like (Fig. 1d)—which ranged in size from 190 to 1235 μm. In manually embedded gels, the initial mixture of 5000 cells per well resulted in an average of 334 ± 66 organoids per well at 7 days and 292 ± 74 organoids per well after 14 days. As noted, the high level of variability resulted in a large discrepancy among the standard deviation values, which further illustrates the difficulty in interpreting experimental findings using traditional embedding techniques.

Using this baseline as our comparator, we next sought to identify the core parameters to reliably generate and guide the formation of organoids using our low-cost bioprinting system (Fig. 1e, f, g) [26]. Our bioprinting method uses CNC processes (Fig. 1g) to guide custom glass capillary microneedles to directly insert cells into 3D locations of polymerized collagen I gels generating a gridded array (Fig. 1e). We have previously shown that these custom glass microneedles impart negligible shear stress on cells and accurately place cells with ranges of 1 to 70 cells with an error rate of less than 10% within less than 1 nL of media [26]. Owing to the non-coring nature of our glass-pulled pipettes, this bioprinting technique confines cell aggregates in specified locations within pre-cast 3D hydrogels following needle extraction and subsequent gel closure, thus eliminating the random cell distribution commonly observed in layer-by-layer processes and manual cell-matrix embedding procedures (Fig. 1e).

**Fig. 2:**
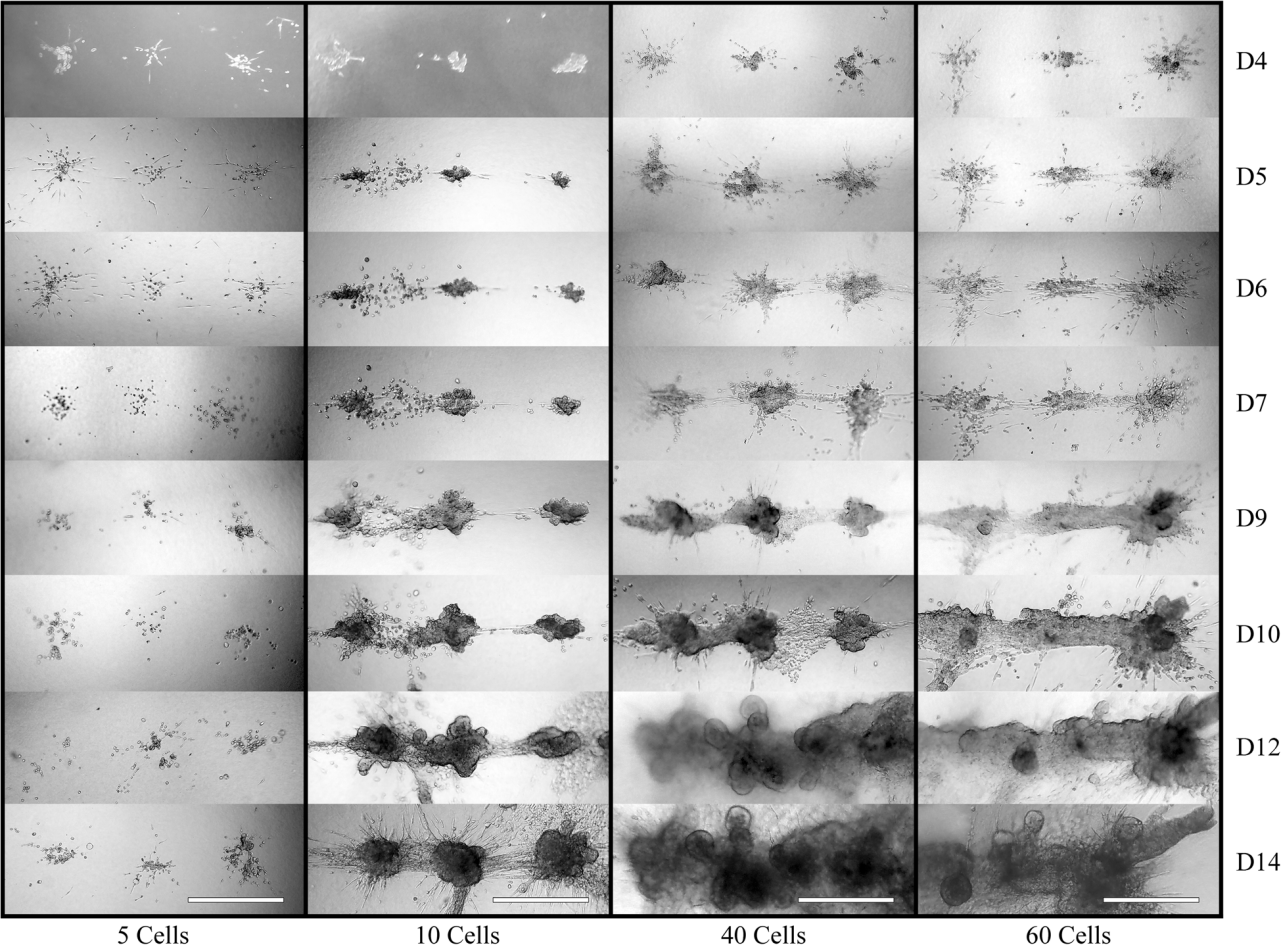
Bioprinting of as few as 10 cells per print location results in consistent organoid formation. MCF12A cells were printed using initial cell concentration of 5, 10, 40, or 60 cells (columns left to right, respectively) at a distance of 500 μm between print locations. Images were taken at days 4, 5, 6, 7, 9, 10, 12, and 14 (rows top to bottom, respectively). Initial injections of 10 or more cells resulted in consistent organoid formation. Consistent fusion of multiple print locations was seen by day 14 when at least 10 cells were printed per injection site. Scale bar: 500 μm

We initially assessed whether the formation frequency of individual human mammary epithelial organoids could be increased by controlling the initial number of singly dissociated cells in a specified location. Using our bioprinting device, we dispensed cell-laden media at equivalent volumes in equally spaced (100 μm) linear arrays inside collagen I gels and tracked them daily for 14 days (Fig. 2).

We found that initial cell injections of not more than 5 (±2) cells formed individual organoids at frequencies of 1 out of 50 and 28 out of 50 at 7 and 14 days, respectively. However, when the initial printed cell number equaled 10 (±3) cells, our system achieved 37 out of 50 and 49 out of 50 organoid efficiency at 7 and 14 days, respectively. Using 40 (±4) and 60 (±5) cells resulted in consistent (50 out of 50) organoid formation for both 7 and 14 days. These results indicate that reliable generation of individual organoids can be achieved by increasing the initial number of cells (≥10) in specified locations with a spacing of 500 μm. We also found that printed cell clusters containing cell numbers of at least 10 consistently develop branched processes exclusively pointed toward neighboring organoids by day 10, forming a contiguous structure. Furthermore, the temporal nature of this branching process increased correlative to the addition of more cells within the initial printing event.

### Directing the generation of large contiguous mammary epithelial organoids

**Fig. 3:**
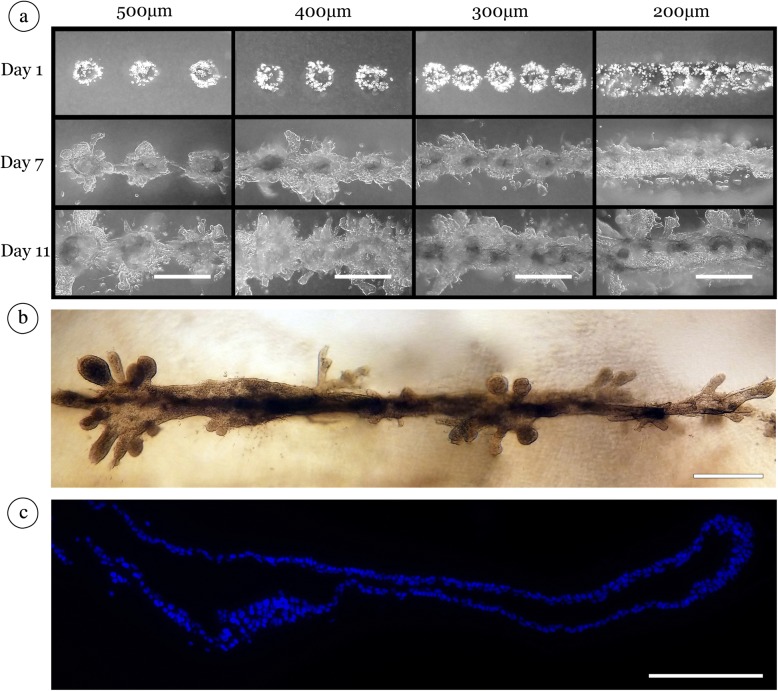
Organoid fusion occurs between organoids printed up to 500 μM apart. (**a**) MCF12A cells were printed (40 cells per print location) at distances of 500 μm, 400 μm, 300 μm, and 200 μm (columns left to right, respectively). Consistent fusion was seen by day 11 in all cases, and contiguous organoids formed between prints spaced not more than 400 μm apart. Scale bar: 500 μm. (**b**) By day 14, organoids fused into contiguous luminal structures that maintained the linear array shape. Scale bar: 500 μm. (**c**) 4′,6-diamidino-2-phenylindole (DAPI) stained 5 μM section of a large contiguous, duct-like structure in 21-day-old MCF12A culture. Scale bar: 200 μm

As the consistent nature of individual organoid formation changed with the variation of cell number, we next sought to determine whether varying distances could evoke a similar impact on the bridged-contiguous organoid formation that we observed in our linear arrays. Specifically, we wished to determine whether organoid spacing could further promote the formation of large-scale contiguous organoids. To this end, we monitored the effect of organoid spacing on growth behavior by printing MCF12A cells along linear arrays of cell deposits containing 40 (± 3) cells in collagen gels (Fig. 3). Data indicated that inter-organoid spacing (≤300 μm) directed collective cell growth of all (36 out of 36) organoids into duct-like patterns along the entire length of the linear array (~4 mm) within 7 days after printing (Fig. 3a). At 400 μm, 34 out of 36 organoids fused within 7 days after printing (Fig. 3a). Twenty-three out of 36 organoids spaced 500 μm apart achieved organoid fusion within 7 days; however, 35 out of 36 of these organoids achieved fusion by day 11 (Fig. 3a). When cells were spaced at at least 700 μm, no fusions (0 out of 36) were seen between printed clusters by day 7. Closer examination of the 500-μm print conditions indicated that cell numbers increased during the first 3 days after printing. We observed formation of coordinated branched extensions directed toward neighboring organoids between days 5 and 7 (Additional file 1: Movie S1). It was noted that decreasing organoid spacing appears to promote the initial formation of a central structure corresponding to the axis of the linear array. This also indicated that individual organoids had a propensity to maintain the linear nature of the initial printed pattern throughout this fusion process (Fig. 3b). Thus, by manipulating the spacing, we can predictably increase the formation of a contiguous structure. This was particularly highlighted where neighboring linear arrays printed at least 700 μm apart were unable to reliably attain directed print geometries within a 14-day observation window. Furthermore, this central structure appears to support the growth of secondary branches, which exclusively radiate from the initial structure (Fig. 3b). DAPI staining of cross-sections of 21-day cultures of MCF12A cells confirmed the presence of contiguous ductal structures resulting from the initial bioprinted cell deposits (Fig. 3c). Of note, some structures were found to have large contiguous lumens. For example, the structure shown in Fig. 3c is over 1 mm in length within a single 5-μm sectional plane. In addition, immunostaining of cross-sections of the resulting structures with antibodies to laminin 1 + 2 and laminin 5 demonstrated basal localization of the laminin proteins, consistent with organoid polarization (Additional file 2: Figure S1a,b). These results indicate that methods described here can direct and promote the formation of hollow ductal structures reminiscent of the morphology of a duct formed *in vivo*.

**Fig. 4:**
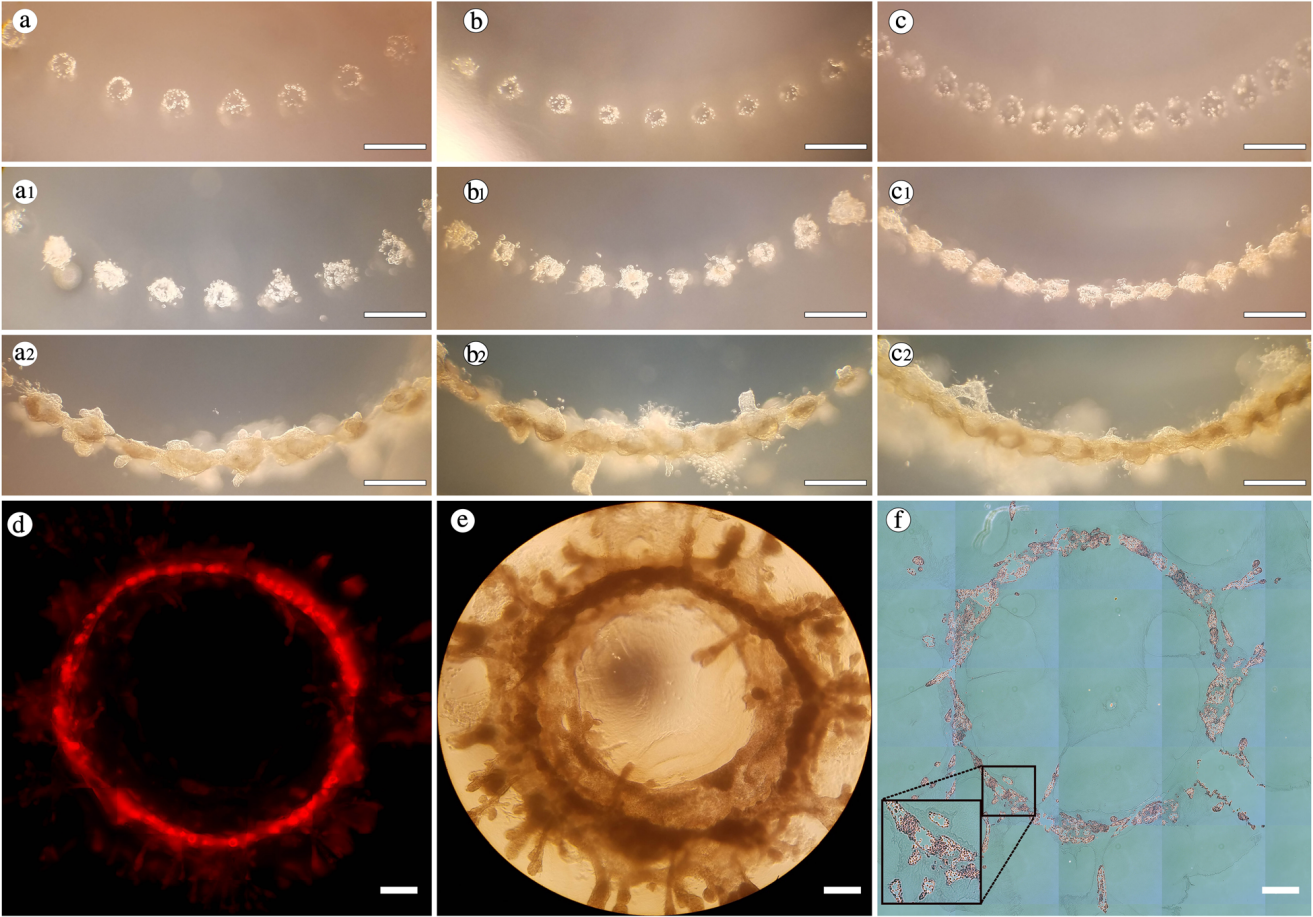
Bioprinting of non-linear organoids. MCF12A cells were printed in 40-cell clusters in a radial pattern with print spacings of (**a**) 500 μm, (**b**) 400 μm, and (**c**) 300 μm. (**a1–c1**) Image of printed cell clusters 7 days after print demonstrating early development into individual organoids. (**a2–c2**) Image of printed cell clusters 14 days after print demonstrating fusion of individual organoids into a contiguous circular organoid. (**d**) Image of red fluorescent protein–positive (RFP^+^) MCF12A cells forming large circular organoid 14 days after print. (**e**) Example of a large circular organoid 24 days after print measuring about 4 mm in diameter. (**f**) Hematoxylin and eosin (H&E) cross-section of circular organoid shown in **e** demonstrating luminal sections within the organoid. Scale bars = 500 μm

To further explore this, we examined the possibility of directing contiguous luminal structures to conform to alternative shapes. We initially printed 40 cell clusters in a radial pattern within rat tail collagen gels with spacing patterns similar to our linear arrays at 500 μm, 400 μm, and 300 μm (Fig. 4a, b, c). After 7 days, all of the print locations had formed individual organoids (Fig. 4a_1_, b_1_, c_1_), and obvious processes and connections were actively forming among the 300-μm spaced injections. By day 14, again similar to our linear arrays, all groups formed contiguous structures which reflected the intended circular geometry (Fig. 4a_2_, b_2_, c_2_, d). Furthermore, our ability to direct mammary epithelial cell (MEC) structures was maintained throughout 24 days of culture, wherein the cells reacted similarly to the linear arrays and maintained the initial print pattern and formed a contiguous luminal circle about 4 mm in diameter (Fig. 4e, f). These findings indicate that mammary epithelial migration patterns are not random but rather that the MCF12A cells actively seek neighboring organoid structures to participate in the formation of large structures (Additional file 1: Movie S1 and Additional file 3: Movie S2). Furthermore, these data clearly highlight the tunable nature of our system, where initial cell number can consistently influence the formation of individual organoids. These data also demonstrate that we can print specific cell numbers with the design of consistently generating large contiguous luminal organoids.

### Cells from 3D printed individual organoids intermingle and form gap junctions with neighboring organoids

**Fig. 5:**
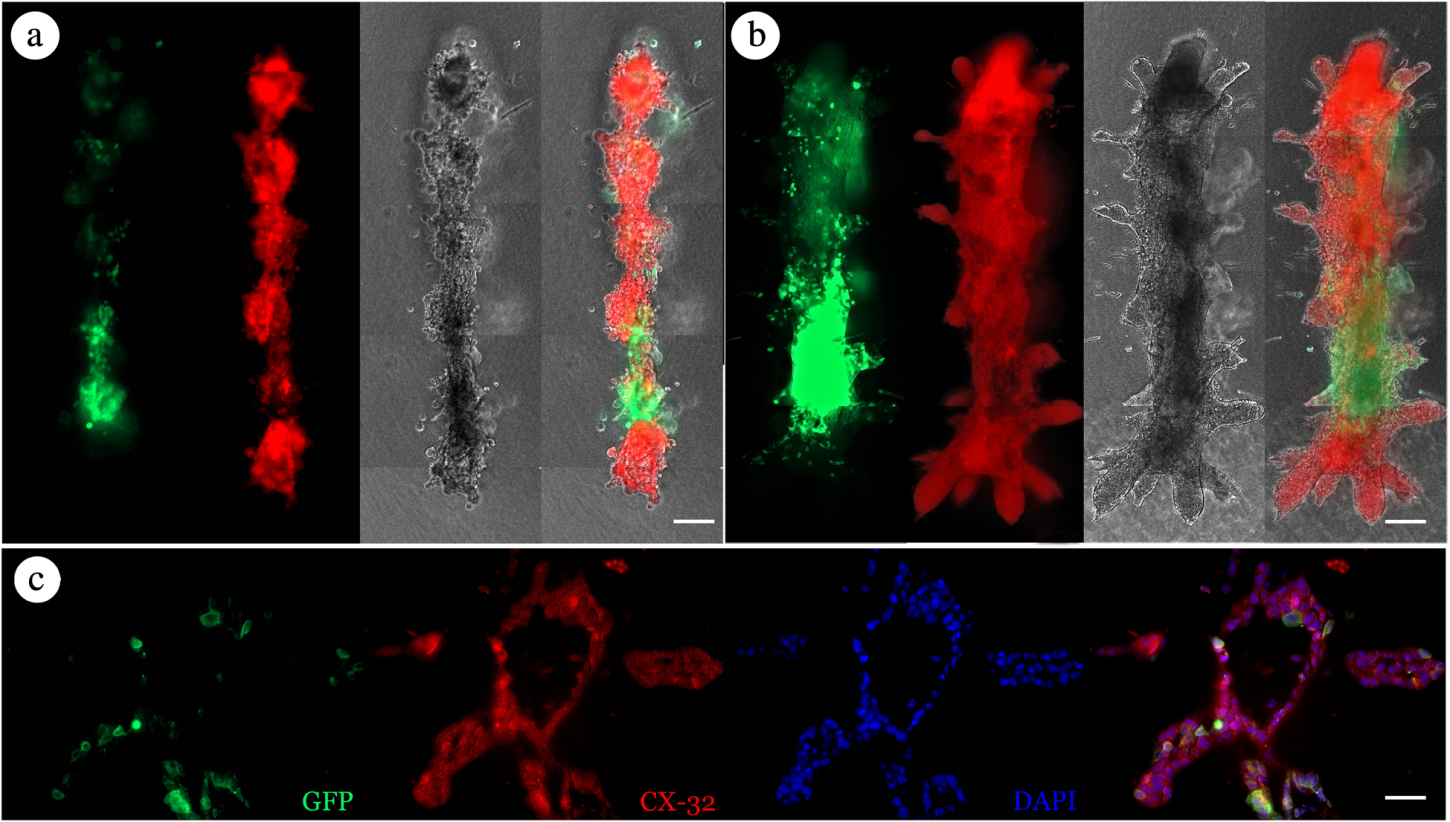
Alternated prints form contiguous organoids with intermingled cells. Alternating red fluorescent protein (RFP)- and green fluorescent protein (GFP)-labeled MCF12A cells. (**a** and **b**) Examples of organoids resulting from alternating prints of RFP^+^ and GFP^+^ MCF12A cells. Forty GFP^+^ and RFP^+^ MCF12A cells were printed with 200-μm spacing, and cells were grown and imaged at (**a**) day 7 and (**b**) day 9. Scale bar: 200 μm. (**c**) Immunofluorescence staining of CX32 (red) and GFP (green) in cross-section of an organoid formed as described in (**a** and **b**). Presence of GFP^+^ cells intermingled along the same lumen as GFP^−^ cells with expression of CX32 along cell boundaries indicates that the cells from adjacent print sites intermingled and formed cellular junctions with cells from neighboring prints to form contiguous structures Scale bars: **a** and **b** = 500 μm; **c** = 50 μm

Having established the cell number and individual organoid spacing necessary to form contiguous organoids within 7 days, we next sought to determine to what extent the individual organoids were integrating with their neighbors. To this end, we printed equally spaced 200-μm linear arrays of alternating RFP- and GFP-labeled MCF12A cells in collagen gels. The RFP and GFP printed cells formed contiguous organoids with a central structure and branched extensions with mixed GFP and RFP cells by day 7 (Fig. 5a). Separating the fluorescent channels of these structures revealed the presence of RFP- and GFP-labeled cells intermingling with one another within regions of the larger structure and branched processes at day 14 (Fig. 5b). Indication of coordinated cellular behavior was further supported by positive staining of gap junction protein connexin-32 (CX32) between RFP and GFP MCF12A cells along the same lumen (Fig. 5c).

### Printing with MCF10A cells and additional sources of ECM

**Fig. 6:**
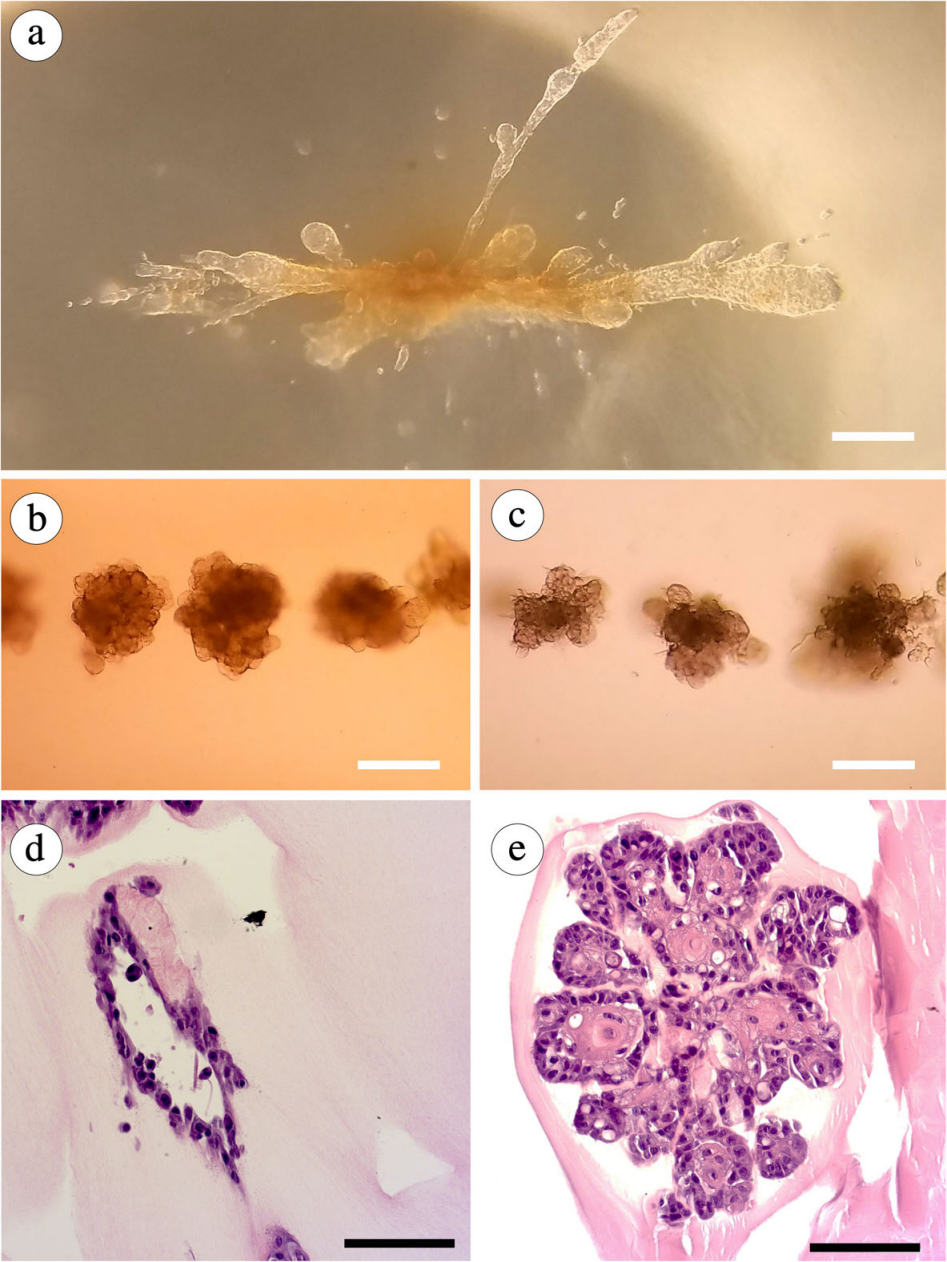
Adaptation of bioprinting protocol for MCF10A cells and Geltrex hydrogels. (**a**) MCF10A cells growing in rat tail collagen 7 days after printing. Scale bar: 250 μm. (**b** and **c**) MCF10A (**b**) and MCF12A (**c**) cell deposits 7 days after print in Geltrex at 500-μm spacing. Scale bar: 250 μm. (**d**) and (**e**) Hematoxylin and eosin (H&E)-stained cross-sections of MCF10A cells 7 days after printing in rat tail collagen (**d**) and Geltrex (**e**). Scale bar: 100 μm


To provide further comparative data to our MCF12A results, we incorporated MCF10A cells, a commonly used cell type in studies of mammary epithelial biology, into our bioprinting methods. When printed into collagen hydrogels via the same protocol described above, the MCF10A cells formed large structures morphologically similar to those seen with MCF12A cells (Fig. 6a; Additional file 4: Movie S3). Next, we evaluated our printing system with the commonly used 3D culture substrate growth-factor reduced Matrigel (Geltrex). Unlike organoid growth in collagen gels, both MCF10A and MCF12A cell types displayed a reduced affinity to undergo organoid fusion to form contiguous structures in Geltrex (Fig. 6b–e; Additional file 5: Movie S4). This is consistent with grown patterns previously reported for Matrigel [29]. However, we also observed in a rare incidence the formation of a large contiguous structure with MCF12As similar to that seen in collagen (not shown). These results demonstrate the versatility of our bioprinting system for *in vitro* culture of mammary epithelial cells in various substrates*.*

Additional file 4: **Movie S3.** MCF10A printed at 500 μm in rat tail collagen hydrogels was followed over 8 days beginning at day 4 after print. Magnification: 10×. (MP4 15854 kb)



Additional file 5: **Movie S4.** MCF10A printed at 500 μm in Geltrex hydrogels was followed over 7 days beginning at day 2 after print. Magnification: 10×. (MP4 11218 kb)


Although rodent-derived collagen models recapitulate many features of human mammary gland biology, interspecies variations in ECM composition, organization, density, and function exist [30]. Furthermore, the use of substrata derived from Engelbreth-Holm-Swarm tumors, commercial preparations, or other non-biomimetic synthetic scaffolds can suffer from batch-to-batch variability. Given our ability to standardize the quantity and spatial distribution of MECs in 3D, we set out to investigate the ability to direct MEC organoid formation in additional sources of ECM by using human-derived collagen gels.

**Fig. 7:**
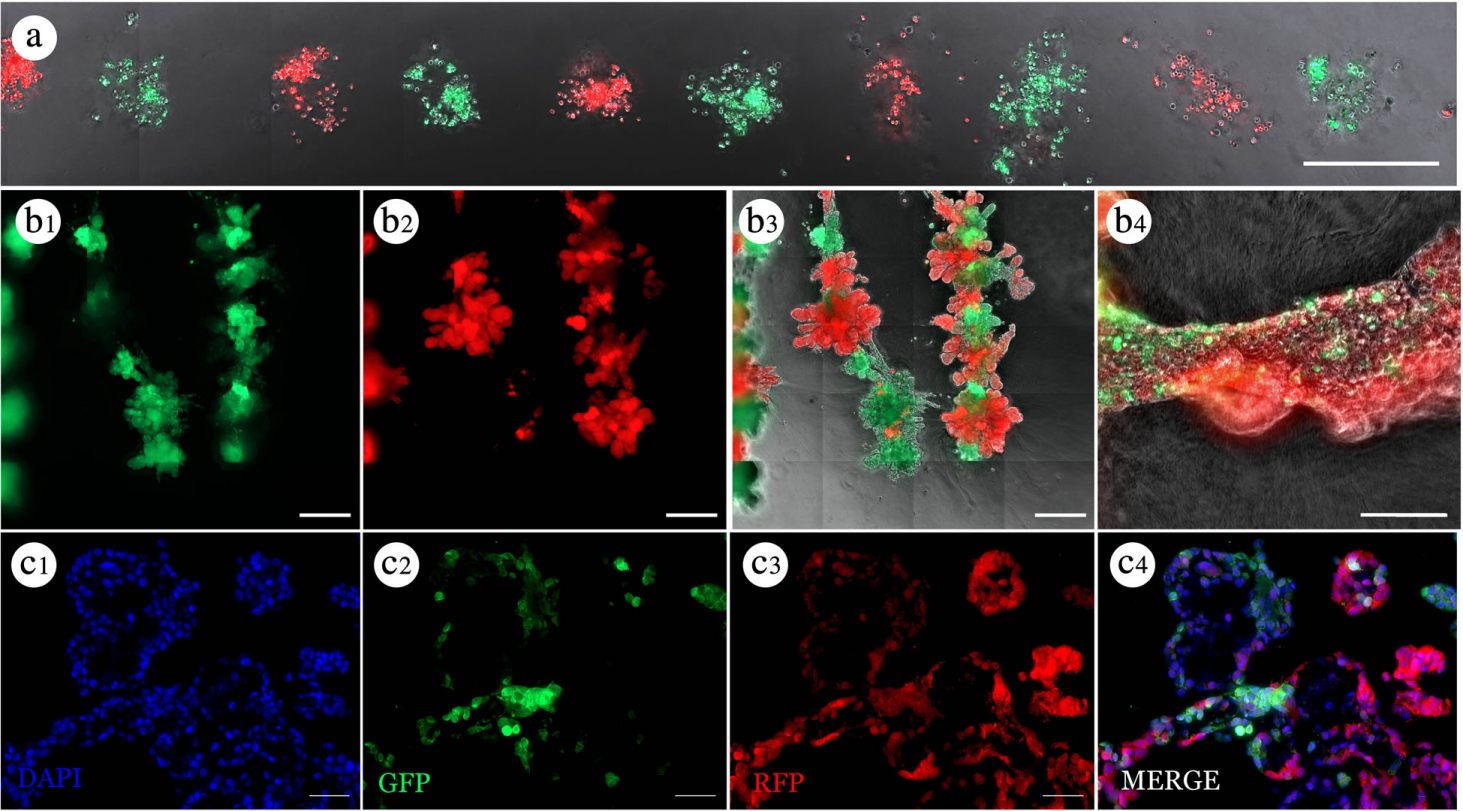
Breast epithelial morphology in human collagen*.* (**a**) Bioprinted red fluorescent protein (RFP) and green fluorescent protein (GFP) MCF12A cell deposits in human collagen gel 2 days after printing. (**b**) GFP (**b1**), RFP (**b2**), and merged (**b3** and **b4**) images of organoid formed from alternating prints of GFP and RFP MCF12A cells in human collagen 7 days after printing; higher magnification image (**b4**) demonstrates mix of GFP^+^ and RFP^+^ cells in the same structure. **c**) Immunofluorescent staining of a cross-section of the organoid in (**b**) with 4′,6-diamidino-2-phenylindole (DAPI) (**c1**; blue), GFP (**c2**; green), and RFP (**c3**; red). Merged image (**c4**) demonstrates presence of both cell types in the same organoid structure. Scale bars: **a** = 200 μm, **b** = 500 μm, **c** = 100 μM

To this end, we again printed RFP- and GFP-labeled MCF12A cells spaced 200 μm apart in linear arrays in human-derived collagen gels (Fig. 7a). After 7 days in culture, both RFP and GFP cells were observed to contribute to the formation of large branched structures (Fig. 7b1–b3). Again, upon closer examination *in situ*, RFP and GFP cells were observed intermingling to form a contiguous organoid structure (Fig. 7b4). Furthermore, data from nine wells, each containing 60 target locations, indicated a total of 528 out of 540 neighboring organoid fusion events within 7 days after printing. Histological staining indicated the presence of GFP- and RFP-labeled cells within the same lumen (Fig. 7c1–c4). These data demonstrate that our bioprinting technique can investigate additional ECM preparations without compromising the number and spatial distribution of printed MECs.

## Discussion

The quest for understanding development and disease in higher organisms has been hindered by a lack of investigative tools to accurately and repeatedly control the many variables that impact 3D *in vitro* model systems. A profound example of this is evidenced from the disparate results from laboratory to laboratory in 3D epithelial organoid systems despite the use of biochemically identical ECM matrices and cell types [18, 21, 22, 31]. In the experiments reported herein, we describe a technique to systematically investigate the extent to which cell–cell and cell–ECM interactions act as regulators of normal epithelial cell differentiation into well-organized structures. By standardizing the number and position of cells inside pre-formed gels, we have developed a method to help standardize the analysis of 3D cultures.

It has been noted that organoids resulting from single, primary epithelial cells vary in morphology and formation efficiency compared with organoids derived from primary epithelial cell clusters [32]. Indeed, we expect that the increase in efficiency in our system was due in large part to our ability to define the quantity of cells being placed initially in close proximity to one another. This observation is a likely cause of much of the high variability seen using manual cell-matrix techniques, where we witnessed individual cells preferentially traveling, not clustering, within the gel. In contrast to this, printed cell clusters were prone to quickly begin internal organization into groups and could collectively seek neighboring organoids (Additional file 1: Movie S1, Additional file 3: Movie S2, Additional file 4: Movie S3 and Additional file 5: Movie S4). Our quantitative data bolstered this idea as the formation frequency of organoids increased significantly when we crossed a critical cell number threshold. Furthermore, our data suggest that reliable control over both initial cell number and organoid spacing permits experimenter-directed fabrication of large-scale branched tubular structures of epithelial origin. These data frame the idea that inter-cellular communications that are received when cells are initially introduced into a foreign 3D environment clearly initiate specific sets of response cascades.

It has been shown that following stable adhesion to ECM components, the mechanical interaction between individual cells and ECM results in the transmission of strain patterns which can extend through hundreds of microns of gel [23, 33, 34]. This applied mechanical strain leads collagen fibers to orient along the direction of the strain [18], which results in increased contact guidance. Furthermore, early studies found a preference for MEC organoids to develop along tension lines between adjacent organoids within collagen gels [35]. In a manner similar to which MECs actively seek neighboring organoids in 3D gels, we find that morphological patterns appear to be associated with the relative position of an individual organoid within the printed array. This may explain our observations that organoids seemed to “sense” their neighboring organoids. We observed that  cells actively traveling between organoids and extending processes preferentially toward each other ultimately lead to organoid fusion. This process allows us to direct organoid growth by manipulating the distances among initial cell deposits.

Throughout the past decade, testing and controlling microenvironmental aspects of 3D culture systems have enabled researchers to bridge the gap between traditional 2D cell culture systems and animal models for studying development and tumorigenesis. We used our bioprinting device to derive a set of guidelines to enable reliable formation of large-scale, human mammary epithelial organoids in 3D hydrogels. These results demonstrate that epithelial organoid morphology can be directed by initial cell-deposit number, spacing, and overall print geometry. However, the development of actual tissues cannot be reduced to cellular events alone. ECM synthesis and assembly in the mammary gland constitute a dynamic and reciprocal relationship between multiple epithelial cell types, myoepithelial cells, adipocytes, endothelial cells, immune cells, and fibroblasts. Where the ECM serves to support and instruct cell behavior, cells also continuously modify and synthesize ECM [30]. The methods described here also demonstrate the capability to accurately deposit multiple cell types as neighboring aggregates, which can communicate and synchronize their structure-forming activities. Our approach allows direct control over the generation of *in vitro* constructs large enough for *in vivo* implantation. More importantly, using this system to investigate co-cultures of two or more cell types in a defined microenvironment would greatly increase the ability to develop reliable 3D surrogate models for breast development and carcinogenesis. This is of particular interest to our group, as we have great interest in understanding how the microenvironment controls differentiation of stem and cancer cells [36–45]. We plan to adapt these protocols for the development of chimeric structures containing cancer and normal epithelial cells as *in vitro* models that mimic our previous *in vivo* findings. Furthermore, we expect the processes outlined here to be easily adaptable to other epithelial cell types, including endothelial cells, to study vascularization and development in other tissue types.

## Conclusions

In summary, these data demonstrate that our CNC-driven 3D bioprinter is capable of repeatedly and reliably printing mammary epithelial structures. Furthermore, through coordinated cluster placement, our system is capable of generating consistent, large contiguous luminal structures. This 3D bioprinter was developed as an open-source project, where we have disseminated the required data/documents for any biological laboratory to manufacture and use. Thus, through digital transfer of G-Code files, these data could easily be replicated in other laboratories.

## Additional files

Additional file 1: **Movie S1.** MCF12A printed at 500 μm was followed over 1 day from day 7. Cells were seen to collectively branch toward neighboring organoids. Magnification: 20×. (M4V 4232 kb)

Additional file 2: **Figure S1.** Polarization of bioprinted structures. Laminin 1 + 2 staining (A; green) and laminin 5 (B; red) of bioprinted MCF12A cells show localization of secreted laminins to the basal layer. Nuclei were counterstained with 4′,6-diamidino-2-phenylindole (DAPI). Scale bars = 50 μM. (JPG 1048 kb)

Additional file 3: **Movie S2.** MCF12A printed at 500 μm was followed over 3 days beginning at day 4 after print. Some individual cells can be seen grouping and traveling between organoids. Magnification: 10×. (M4V 2860 kb)

## Abbreviations

2D: Two-dimensional
3D: Three-dimensional
CNC: Computer numerical control
CO_2_: Carbon dioxide
CX32: Connexin 32
DAPI: 4′,6-diamidino-2-phenylindole
ECM: Extracellular matrix
GFP: Green fluorescent protein
MEC: Mammary epithelial cell
RFP: Red fluorescent protein
