# Correction to: Consistent and reproducible cultures of large-scale 3D mammary epithelial structures using an accessible bioprinting platform

**DOI:** 10.1186/s13058-018-1069-9

**Published:** 2018-11-19

**Authors:** John A. Reid, Peter A. Mollica, Robert D. Bruno, Patrick C. Sachs

**Affiliations:** 1 Biomedical Engineering Institute, College of Engineering, Old Dominion University, 5115 Hampton Blvd, Norfolk, VA 23529 USA; 2 School of Medical Diagnostic & Translational Sciences, College of Health Sciences, Old Dominion University, 5115 Hampton Blvd, Norfolk, VA 23529 USA

## Correction

Following publication of the original article [1], the authors reported a typesetting error in the spelling of the second author’s name.

Incorrect: Peter M. Mollica^2^

Correct: Peter A. Mollica^2^

The publishers apologise for this error. The original article [1] has been updated.
