# Supplementary figures and images for: Consistent and reproducible cultures of large-scale 3D mammary epithelial structures using an accessible bioprinting platform
Source: Breast Cancer Res. 2018 Oct 10;20:122. doi: 10.1186/s13058-018-1045-4 (PMC6180647; doi:10.1186/s13058-018-1045-4)

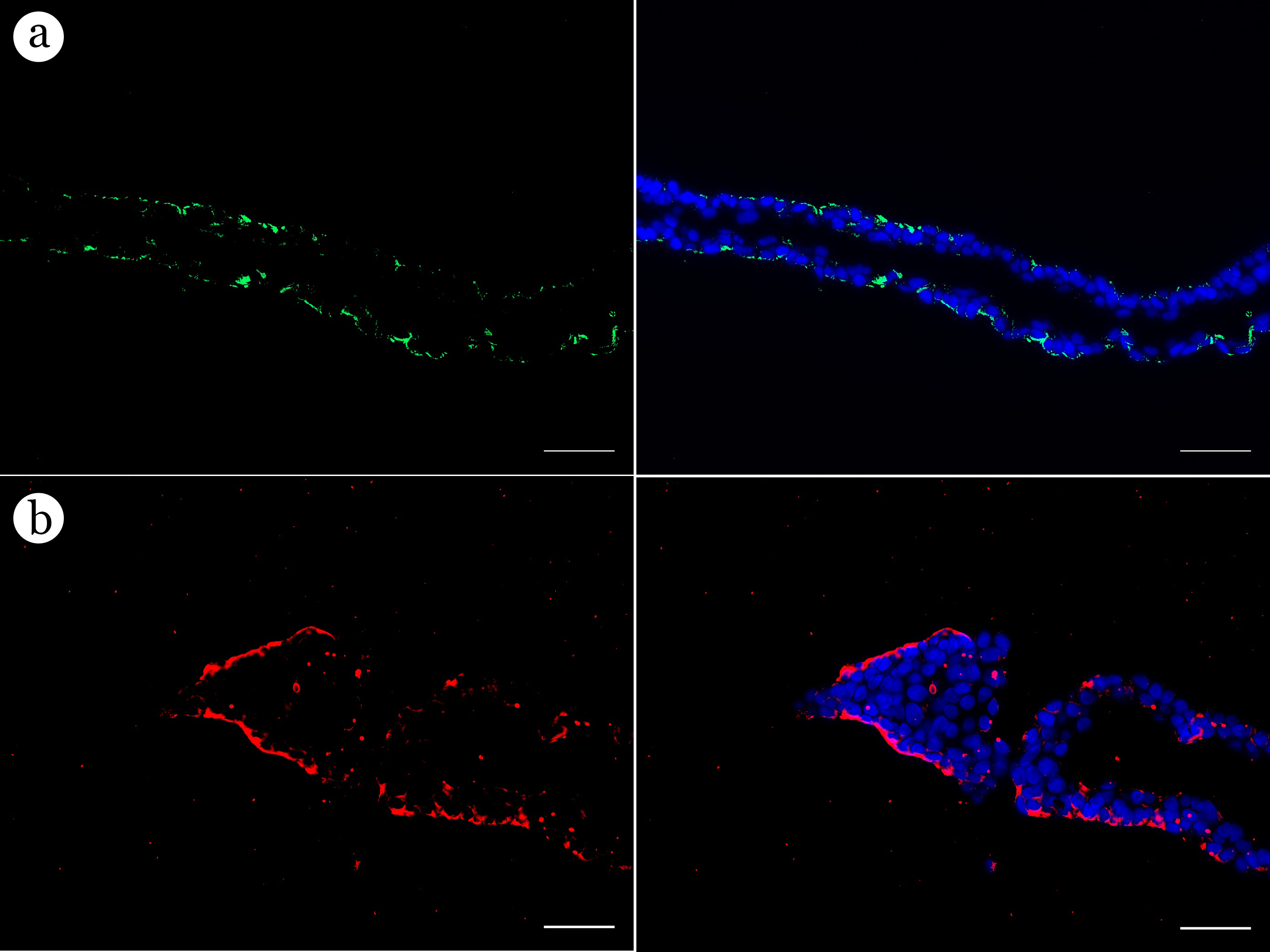

Supplement: Supplementary file 4 — Figure S1. Polarization of bioprinted structures. Laminin 1 + 2 staining (A; green) and laminin 5 (B; red) of bioprinted MCF12A cells show localization of secreted laminins to the basal layer. Nuclei were counterstained with 4′,6-diamidino-2-phenylindole (DAPI). Scale bars = 50 μM. (JPG 1048 kb) [file 13058_2018_1045_MOESM2_ESM.jpg]
